# Comprehensive characterization of cell disulfidptosis in human cancers: An integrated pan-cancer analysis

**DOI:** 10.1016/j.gendis.2023.101095

**Published:** 2023-09-14

**Authors:** Guo Zhao, Yale Jiang, Yuning Wang, Shuhang Wang, Ning Li

**Affiliations:** Clinical Trial Center, National Cancer Center/National Clinical Research Center for Cancer/Cancer Hospital, Chinese Academy of Medical Sciences and Peking Union Medical College, Beijing 100021, China

Excessive accumulation of disulfide molecules, like cystine, can induce disulfide stress and high toxicity in cells, but the reduced form of nicotinamide adenine dinucleotide phosphate can reverse this process and mitigate disulfide stress.^1^^,^^2^ One previous study showed that aberrant expression of cystine transporters, such as solute carrier family 7 member 11 (SLC7A11), or excessive cystine uptake, combined with glucose deprivation, could rapidly deplete the nicotinamide adenine dinucleotide phosphate level, leading to excessive disulfide accumulation and subsequent cell death.^1^ However, the underlying mechanism is unclear. Recently, Liu and colleagues reported a novel cell death pattern based on disulfide stress, termed “disulfidptosis”, which is distinct from common forms of cell death, including autophagy, apoptosis, and ferroptosis.^3^ However, so far, no study has been conducted to analyze the roles of disulfidptosis genes in pan-cancer. Insights into disulfidptosis genes in pan-cancers are crucial to clarify disulfidptosis involved tumorigenesis and to develop inhibitors targeting disulfidptosis with a clinical potential as cancer treatment.^4^

In our study, to investigate the mechanisms of disulfidptosis-related tumors and provide new ideas for tumor treatment by controlling disulfidptosis, we identified a total of 10 disulfidptosis-related genes, including six pro-disulfidptosis genes (*NUBPL*, *NDUFA11*, *LRPPRC*, *OXSM*, *NDUFS1*, and *GYS1*) and four anti-disulfidptosis genes (*SLC7A11*, *SLC3A2*, *RPN1*, and *NCKAP1*).^3^ Then, we performed a systematic pan-cancer analysis of 10 disulfidptosis-related genes across 32 types of cancer through multi-omic profiling data. We comprehensively analyzed the genetic alternation and characterized the expression profile of disulfidptosis-related genes in pan-cancer using multiple public databases. Furthermore, we evaluated the potential association between disulfidptosis gene expression and tumor microenvironment or tumor stemness. Besides, we also constructed an interaction network of disulfidptosis genes and predicted the potential drugs for targeting the disulfidptosis pathway. Details of these analyses are provided in the Supplementary Information.

Genetic alternations, such as copy number variations (CNVs) and single nucleotide variants (SNVs), are associated with tumor occurrence, progression, and treatment response.^5^ Figure S1A showed that *LRPPRC* was the most frequently mutated disulfidptosis gene in 32 human cancers, especially in uterine corpus endometrial carcinoma (UCEC, 34%) and skin cutaneous melanoma (SKCM, 16%), both of which had a mutation rate of over 15%. In UCEC, all 10 disulfidptosis genes were screened for SNVs. UCEC had a higher mutation rate in all disulfidptosis genes compared with other cancers. Figure S2 showed that some disulfidptosis genes that have relatively high mutation rates (over 15%) could lead to the activation or inhibition of corresponding proteins in UCEC and SKCM. Notably, 34% and 16% mutation rates of LRPPRC are found in UCEC and SKCM, respectively and our analysis showed that the mutations of LRPPRC could lead to activation of the protein in UCEC, while inhibition of the protein in SKCM (Fig. S2). The SNV landscape revealed that SNV changes in disulfidptosis genes occurred in all 570 tumor patients, with a frequency of 100% and missense mutation as the major type of SNV (Fig. S1B). The profile of disulfidptosis gene SNVs associated with survival showed that kidney renal papillary cell carcinoma and liver hepatocellular carcinoma were the most likely to be associated with disulfidptosis gene SNVs with a strongly positive association in progression-free survival and disease-free interval. However, only a few genes were significant in these cancers, with most of the other cancer types revealing no significant difference (Fig. S1C). Furthermore, CNV of the disulfidptosis genes had multiple function modes in pan-cancers. The pan-cancer pie chart of the disulfidptosis gene set revealed that most CNVs were of the heterozygous type, with only a small percentage of homozygous CNVs (Fig. S1D). Therefore, the transcriptional dysregulation of disulfidptosis genes in pan-cancers is mainly due to heterozygous deletion or amplification. Besides, the mRNA expression of most disulfidptosis genes was positively associated with CNV level, in addition to SLC7A11 in kidney renal papillary cell carcinoma (Fig. S1E). Additionally, UCEC was the top cancer type that was most closely to be associated with the CNV level of disulfidptosis genes. However, for the other cancer types, only a few disulfidptosis genes were significant (Fig. S1F). The heterozygous CNV of disulfidptosis genes could affect the tumorigenesis and prognosis of UCEC. In these cancers, disulfidptosis gene CNVs were associated with the survival of patients. Meanwhile, methylation of disulfidptosis genes differed in a few cancer types (Fig. S1G). Some cancer types, such as lung squamous cell carcinoma, were significantly correlated with mRNA expression for some disulfidptosis genes and methylation, but most of them were negative (Fig. S1H). These results indicated that hypermethylation may be one of the underlying mechanisms of down-regulation of some disulfidptosis genes in cancers.

Among the 10 disulfidptosis genes, *RPN1* and *SLC3A2* were highly expressed, *GYS1* and *LRPPRC* were moderately expressed, and the other genes were lowly expressed in pan-cancers (Fig. 1A). The expression of disulfidptosis genes found that kidney renal clear cell carcinoma (KIRC) had the most significantly different genes. Besides *NDUFA11*, the other disulfidptosis genes were significantly altered in KIRC (Fig. 1B). We also verified our results by combining three databases, including TCGA, GTEx, and TARGET cohorts. All 10 disulfidptosis genes were significantly dysregulated in KIRC (Fig. S3). *LRPPRC* and *NDUFS1* genes showed the most significant positive correlation (Fig. 1C), whereas *NDUA11* and *NCKAP1* showed the most significant negative correlation (Fig. 1C). One gene may have a disparate expression mode in the different cancer subtypes. Subtype analysis showed that expression of disulfidptosis genes showed significant relevance in some cancer subtypes, like breast cancer (Fig. 1D). As shown in Figure 1D, the disulfidptosis genes were also closely associated with the activation or inhibition of various tumorigenesis-related pathways. Based on these results, a pathway regulation network (Fig. S6A) and microRNA-mRNA regulation network of disulfidptosis genes were constructed. Seven of the ten disulfidptosis genes were identified to be regulated by microRNAs. A larger number of microRNAs may be involved in the expression regulation of the disulfidptosis genes, making up the complex miRNA-mRNA regulation network (Fig. S6B). These results provided insights into the potential cross-talks between disulfidptosis and other signaling pathways or non-coding RNAs. Meanwhile, the survival for KIRC had the highest correlation with the expression of disulfidptosis genes. The high expression of three pro-disulfidptosis genes (*NUBPL*, *LRPPRC*, and *NDUFS1*) and the anti-disulfidptosis gene *NCKAP1* were associated with a lower risk of KIRC in patients. We further performed prognosis analysis based on TCGA, GTEx, and TARGET cohorts and found that these 10 genes are closely associated with cancer prognosis (Fig. S4).

**Figure 1:**
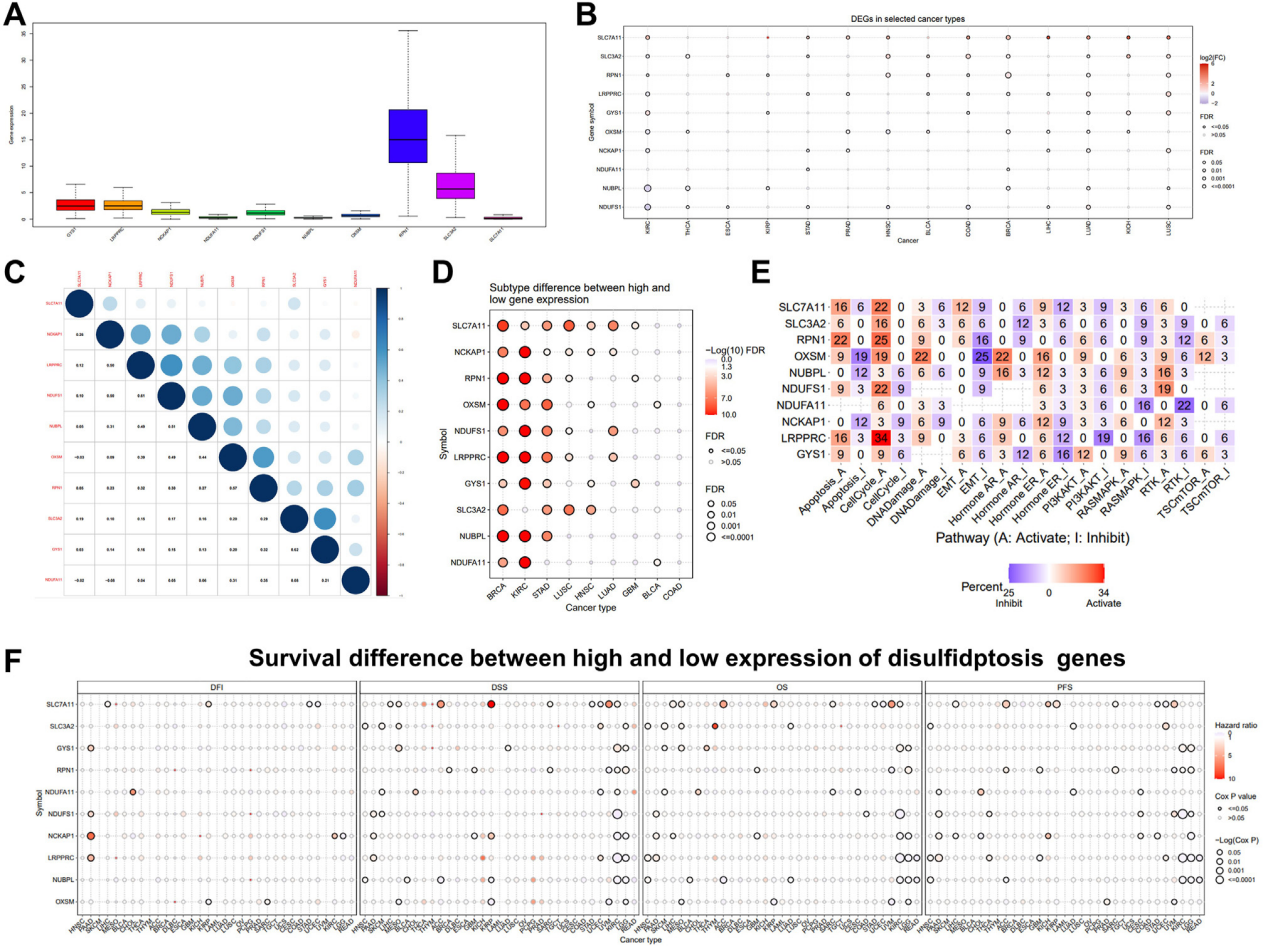
Pan-cancer expression and cross-talk profiles of disulfidptosis gene set. **(A)** Increased or decreased expression of disulfidptosis genes in cancers. **(B)** Expression difference between cancer and non-cancer tissues from TCGA. **(C)** The correlation between the disulfidptosis genes. The blue dots indicate positive correlation and the red dots indicate negative correlation. **(D)** Expression differences between subtypes of cancers. **(E)** Heatmap of the percentage of the effect of disulfidptosis genes on other cancer pathway activity. **(F)** Survival difference between the high and low expression of the disulfidptosis gene.

To further explore the association between disulfidptosis genes and tumor microenvironment, tumor stemness was analyzed in all types of cancer. The correlation analysis of tumor microenvironment revealed a negative association between the expression of all disulfidptosis genes and the immune score of pancreatic adenocarcinoma, and between *LRPPRC* expression and the immune score of nearly all cancers (Fig. S5A). Besides, *GYS1* and *RPN1* expression were positively associated with the stromal score of brain lower-grade glioma (Fig. S5B). Further correlation analysis indicated that RNAss was negatively correlated with *NCKAP1* expression and positively correlated with *LRPPRC* expression in pan-caner (Fig. S5C). The expression of disulfidptosis genes was negatively associated with DNAss in cholangiocarcinoma but positively associated with DNAss in ovarian cancer (Fig. S5D). We also analyzed the potential correlation between disulfidptosis gene expression and drug sensitivity in multiple human cancer cell lines. The results suggested that *GYS1* expression was negatively associated with drug sensitivity to many chemotherapy agents, including dasatinib, temsirolimus, and AZD6482 (Fig. S6C). Meanwhile, *NCKAP1* and *SLC7A11* expression were positively associated with drug sensitivity to ABT-737, olaparib, and belinostat (Fig. S6D).

In conclusion, our study revealed that the expression alterations of disulfidptosis genes might be involved in the activation or inhibition of various cancer-related pathways. Meanwhile, our results also highlight that disulfidptosis genes are closely associated with tumor microenvironment and tumor stemness, which may affect patient response to therapy. Importantly, we analyzed the correlations between disulfidptosis and drug sensitivity and found that the disulfidptosis pathway may be crucial in reversing drug resistance. With the increasing interest in disulfidptosis, like ferroptosis in cancer research, these in-time profile analyses will provide new ideas and useful information for future studies on the potential of disulfidptosis as a treatment strategy in various cancers.

## Author contributions

Study concept and design: NL and SW. Data acquisition and data cleaning: GZ. Data analysis and interpretation: GZ, YJ, and YW. Manuscript drafting: GZ, YJ, and YW. Supervision: LM and SZ. Proofreading and revision: NL and SW. Critical revision of the manuscript for important intellectual content: All authors. The authors read and approved the final manuscript.

## Conflict of interests

The authors declare that they have no competing interests.

## Funding

This work was supported by the National Natural Science Foundation of China (No. 82272951, 82272953), Beijing Municipal Health Commission (Beijing Demonstration Research Ward BCRW20200303), and Chinese Academy of Medical Sciences (No. 2022-I2M-C&T-B-070).

## Data availability

Publicly available datasets were analyzed in this study. These data can be found at: https://portal.gdc.cancer.gov/. Please contact the corresponding author for further data requests.

## Appendix A Supplementary data

Multimedia component 1 Figure S1 Pan-cancer genetic profiles of the disulfidptosis gene set. **(A)** Heatmap of the mutation frequency. The numbers represent the number of samples that have the corresponding mutated gene for a given cancer. "0" indicates that there was no mutation in the gene coding region, the blank indicates there was no mutation in any region of the gene, and the color represents the mutation frequency. **(B)** The SNV landscaped plot of disulfidptosis genes in pan-cancer. **(C)** Survival difference between CNV and wild type of each disulfidptosis gene. **(D)** CNV distribution pie chart across cancers. Hete Amp represents heterozygous amplification; Hete Del represents heterozygous deletion; Homo Amp represents homozygous amplification; Homo Del represents homozygous deletion; None means no CNV. **(E)** The correlation between CNV and mRNA expression. **(F)** Survival difference between CNV groups. **(G)** Methylation differences between tumor and normal tissues. **(H)** The correlation between methylation and mRNA expression. The red represents the positive correlation, while the blue represents the negative correlation. Figure S2 The protein expression of disulfidptosis genes that have an over 15% mutation rate. **(A)** LRPPRC in UCEC, **(B)** NCKAP1 in UCEC, **(C)** NDUFS1 in UCEC, **(D)** SLC3A2 in UCEC, **(E)** GYS1 in UCEC, **(F)** SLC7A11 in UCEC, **(G)** OXSM in UCEC, **(H)** LRPPRC in SKCM, and **(I)** SLC7A11 in SKCM. Figure S3 The RNA expression of **(A)** SLC7A11, **(B)** NCKAP1, **(C)** LRPPRC, **(D)** NDUFS1, **(E)** NUBP1, **(F)** OXSM, **(G)** RPN1, **(H)** SLC3A2, **(I)** GYS1, and **(J)** NDUFA11 in various cancer types and paired normal tissues. ACC, adrenocortical carcinoma; ALL, acute lymphoblastic leukemia; BLCA, bladder urothelial carcinoma; BRCA, breast invasive carcinoma; CHOL, cholangiocarcinoma; COAD, colon adenocarcinoma; READ, rectum adenocarcinoma; COADREAD, colon adenocarcinoma and rectum adenocarcinoma; DLBC, diffuse large B-cell lymphoma; ESCA, esophageal carcinoma; GBM, glioblastoma multiforme; HNSC, head and neck squamous cell carcinoma); GBMLGG, glioma; KIPAN, pan-kidney cohort (KICH + KIRC + KIRP); KICH, kidney chromophobe; KIRC, kidney renal clear cell carcinoma; KIRP, kidney renal papillary cell carcinoma; AML, acute myeloid leukemia; LGG, brain lower-grade glioma; LIHC, liver hepatocellular carcinoma; LUAD, lung adenocarcinoma; LUSC, lung squamous cell carcinoma; MESO, mesothelioma; OV, ovarian cancer; PAAD, pancreatic adenocarcinoma; PCPG, pheochromocytoma and paraganglioma; PRAD, prostate adenocarcinoma; SARC, sarcoma; SKCM, skin cutaneous melanoma; STES, stomach and esophageal carcinoma; TGCT, testicular germ cell tumor; THCA, thyroid carcinoma; THYM, thymoma; UCEC, uterine corpus endometrial carcinoma; UCS, uterine carcinosarcoma; UVM, uveal melanoma. Figure S4 The prognostic value of **(A)** SLC7A11, **(B)** NCKAP1, **(C)** LRPPRC, **(D)** NDUFS1, **(E)** NUBP1, **(F)** OXSM, **(G)** RPN1, **(H)** SLC3A2, **(I)** GYS1, and **(J)** NDUFA11 in various cancer types. Figure S5 Correlation of disulfidptosis gene expression with tumor microenvironment and tumor stemness in pan-cancer. **(A, B)** Disulfidptosis gene expression associated with immune score and stromal score in diverse cancers. The red dots indicate a positive correlation between gene expression in the tumor and immune or stromal score, and the green dots indicate a negative correlation. **(C, D)** Disulfidptosis gene expression associated with RNAss and DNAss in different cancers. The red dots indicate a positive correlation between gene expression in the tumor and stemness score, and the blue dots indicate a negative correlation. RNAss, RNA stemness score; DNAss, DNA stemness score. The red represents the positive correlation, while the blue represents the negative correlation. Figure S6 The cross-talk profile and drug sensitivity analysis of the disulfidptosis gene set. **(A)** Pathway regulation network of disulfidptosis genes. A line represents a connection between genes and different pathways, where a solid line represents activation, and a dashed line represents inhibition. The color of the line represents different cancer types. **(B)** The microRNA (miRNA) network of disulfidptosis genes. A miRNA and one regulator connection node represent miRNA regulation of a gene. Node size is positively correlated with the node's degree, and edge width is defined by the absolute value of the correlation coefficient. **(C)** The correlation between GDSC drug sensitivity and disulfidptosis gene expression. **(D)** The correlation between CTRP drug sensitivity and disulfidptosis gene expression. The red represents the positive correlation, while the blue represents the negative correlation. Multimedia component 1

## References

[1] Liu X., Olszewski K., Zhang Y. (2020). Cystine transporter regulation of pentose phosphate pathway dependency and disulfide stress exposes a targetable metabolic vulnerability in cancer. Nat Cell Biol.

[2] Joly J.H., Delfarah A., Phung P.S., Parrish S., Graham N.A. (2020). A synthetic lethal drug combination mimics glucose deprivation-induced cancer cell death in the presence of glucose. J Biol Chem.

[3] Liu X., Nie L., Zhang Y. (2023). Actin cytoskeleton vulnerability to disulfide stress mediates disulfidptosis. Nat Cell Biol.

[4] Tong X., Tang R., Xiao M. (2022). Targeting cell death pathways for cancer therapy: recent developments in necroptosis, pyroptosis, ferroptosis, and cuproptosis research. J Hematol Oncol.

[5] Á Bartha, Győrffy B. (2019). Comprehensive outline of whole exome sequencing data analysis tools available in clinical oncology. Cancers.

