# Supplementary material for: Comprehensive characterization of cell disulfidptosis in human cancers: An integrated pan-cancer analysis
Source: Genes Dis. 2023 Sep 14;11(4):101095. doi: 10.1016/j.gendis.2023.101095 (PMC10865245; doi:10.1016/j.gendis.2023.101095)
Supplement: Multimedia component 2 [file mmc2.docx]

**Methods and Materials**

**Data collection and download**

The expression, single-nucleotide variant (SNV), copy number variant (CNV), and methylation, data with clinical information were collected and downloaded from The Cancer Genome Atlas (TCGA) [1]. The miRNA regulation data were collected from databases including experimentally verified (scientific papers, TarBase [2], miRTarBase [3], and mir2disease [[4]]) data, and targetscan and miRanda predicted data. The immune therapy survival data were accessed from the Tumor Immune Dysfunction and Exclusion (TIDE) [5]. Besides, The Genotype-Tissue Expression (GTEx) (https://gtexportal.org/home/index.html) were used to download the normal tissues as paracancerous tissues. And Therapeutically Applicable Research to Generate Effective Treatments (TARGET) (<https://www.cancer.gov/ccg/research/genome-sequencing/target>) was used to verify the prognostic value of disulfidptosis in pan-cancer.

**Genetic alterations and expression analysis**

All the expression and methylation analyses and plotting were implemented by R software and ggplot2 or accessed from the website of the data source. SNV plots were generated by the maftools [6]. CNV data were processed with GISTICS2.0 [7]. Data were analyzed using the t-test or ANOVA t-test. To compare the data, the integrated level of the expression of the disulfidptosis gene set was calculated using the gene set variation analysis (GSVA) with the R package GSVA [8]. The protein expression of disulfidptosis genes were performed through OSppc website (https://bioinfo.henu.edu.cn/Protein/OSppc.html).

**Survival analysis**

The mRNA expression, methylation, and clinical survival data were analyzed. Tumor samples were divided into high and low groups according to the median gene RSEM value. The R package survival was used to fit the survival time and survival status for the two groups. A Cox Proportional-Hazards model was used to calculate survival risk (Hazard ratio, HR) for every gene. A log-rank test of Kaplan-Meier survival was performed for each gene.

**Pathway activity** **and microRNA regulation network analysis**

Reverse-phase protein array (RPPA) data from the TCPA database were used to calculate scores for 7876 samples [9]. Ten cancer-related pathways included tuberous sclerosis 1 protein (TSC)/mechanistic target of rapamycin (mTOR), receptor tyrosine kinase (RTK), phosphatidylinositol-4,5-bisphosphate-3-kinase (PI3K)/protein kinase B (AKT), RAS/mitogen-activated protein kinase (MAPK), hormone estrogen receptor (ER), hormone androgen receptor (AR), epithelial-mesenchyme transition (EMT), the DNA Damage Response, cell cycle, apoptosis pathways. The pathway score is the sum of the relative protein level of all positive regulatory components minus that of negative regulatory components in a particular pathway. The pathway activity score (PAS) was estimated as in previous studies [10]; gene expression was divided into two groups (High and Low) by the median expression and the difference in PAS between groups was analyzed using Student’s t-test where the P-value was adjusted by the FDR. The FDR ≤0.05 was considered significant. When PAS (Gene A group High) >PAS (Gene A group Low), gene A was considered to have an activating effect on this pathway; otherwise, it had an inhibitory effect on the pathway. Only miRNA-gene pairs that have recorded data were used to calculate the expression correlation. The miRNA expression and gene expression were merged via the TCGA barcode. The association between paired mRNA and miRNA expression was tested based on a Pearson product-moment correlation coefficient and the t-distribution. The P-value was adjusted by the FDR and only significant connections were plotted. Correlations were calculated for all paired samples. Meanwhile, in consideration of the presence of positive regulators, including transcription factors, a miRNA-gene pair with a negative correlation will be considered as a potential negatively regulated pair. The network was constructed using the visNetwork R package.

**Tumor microenvironment, stemness and drug sensitivity analysis**

Stromal and immune cell scores were calculated by using the ESTIMATE algorithm in R-package “estimate” and “limma” [11] for calculating the presence of infiltrating stromal/immune cells in pan-cancer tissues (using disulfidptosis gene expression profiles). A correlation analysis between disulfidptosis gene expression and RNA stemness score (RNAss), DNA stemness score (DNAss) was performed through the Spearman’s method, using the “cor. Test” command and R package “limma.” Both indicators were visualized by the R-package “corrplot.” Correlation analysis of HER family genes expression with the tumor immune microenvironment and stemness score in selected cancer was pursued by applying the R-package “reshape2”, “ggpubr”, “ggplot2” and “limma”. Drug sensitivity processed data was downloaded from the Genomics of Drug Sensitivity in Cancer (GDSC) [12] and Cancer Therapeutics Response Portal (CTRP) database [13]. Data processing and result visualization use R package “impute”, “limma”, “ggplot2”, and “ggpubr”.

**Statistical analysis**

All statistical analyses were performed using the R software. A Cox proportional hazards model was used to calculate survival risk and hazard ratio (HR). Correlation analysis was performed using the Spearman correlation test. Group comparisons were analyzed using T. test or ANOVA t-test. The prognostic significance of every variable was estimated using Kaplan-Meier survival curves and compared using log-rank tests. P-value <0.05 was considered statistically significant

**References**

1. Cancer Genome Atlas Research N, Weinstein JN, Collisson EA, Mills GB, Shaw KR, Ozenberger BA, Ellrott K, Shmulevich I, Sander C, Stuart JM: **The Cancer Genome Atlas Pan-Cancer analysis project**. *Nat Genet* 2013, **45**(10):1113-1120.

2. Karagkouni D, Paraskevopoulou MD, Chatzopoulos S, Vlachos IS, Tastsoglou S, Kanellos I, Papadimitriou D, Kavakiotis I, Maniou S, Skoufos G *et al*: **DIANA-TarBase v8: a decade-long collection of experimentally supported miRNA-gene interactions**. *Nucleic Acids Res* 2018, **46**(D1):D239-D245.

3. Huang HY, Lin YC, Li J, Huang KY, Shrestha S, Hong HC, Tang Y, Chen YG, Jin CN, Yu Y *et al*: **miRTarBase 2020: updates to the experimentally validated microRNA-target interaction database**. *Nucleic Acids Res* 2020, **48**(D1):D148-D154.

4. Jiang Q, Wang Y, Hao Y, Juan L, Teng M, Zhang X, Li M, Wang G, Liu Y: **miR2Disease: a manually curated database for microRNA deregulation in human disease**. *Nucleic Acids Res* 2009, **37**(Database issue):D98-104.

5. Fu J, Li K, Zhang W, Wan C, Zhang J, Jiang P, Liu XS: **Large-scale public data reuse to model immunotherapy response and resistance**. *Genome Med* 2020, **12**(1):21.

6. Mayakonda A, Lin DC, Assenov Y, Plass C, Koeffler HP: **Maftools: efficient and comprehensive analysis of somatic variants in cancer**. *Genome Res* 2018, **28**(11):1747-1756.

7. Mermel CH, Schumacher SE, Hill B, Meyerson ML, Beroukhim R, Getz G: **GISTIC2.0 facilitates sensitive and confident localization of the targets of focal somatic copy-number alteration in human cancers**. *Genome Biol* 2011, **12**(4):R41.

8. Hanzelmann S, Castelo R, Guinney J: **GSVA: gene set variation analysis for microarray and RNA-seq data**. *BMC Bioinformatics* 2013, **14**:7.

9. Li J, Lu Y, Akbani R, Ju Z, Roebuck PL, Liu W, Yang JY, Broom BM, Verhaak RG, Kane DW *et al*: **TCPA: a resource for cancer functional proteomics data**. *Nat Methods* 2013, **10**(11):1046-1047.

10. Akbani R, Ng PK, Werner HM, Shahmoradgoli M, Zhang F, Ju Z, Liu W, Yang JY, Yoshihara K, Li J *et al*: **A pan-cancer proteomic perspective on The Cancer Genome Atlas**. *Nat Commun* 2014, **5**:3887.

11. Diboun I, Wernisch L, Orengo CA, Koltzenburg M: **Microarray analysis after RNA amplification can detect pronounced differences in gene expression using limma**. *BMC Genomics* 2006, **7**:252.

12. Yang W, Soares J, Greninger P, Edelman EJ, Lightfoot H, Forbes S, Bindal N, Beare D, Smith JA, Thompson IR *et al*: **Genomics of Drug Sensitivity in Cancer (GDSC): a resource for therapeutic biomarker discovery in cancer cells**. *Nucleic Acids Res* 2013, **41**(Database issue):D955-961.

13. Rees MG, Seashore-Ludlow B, Cheah JH, Adams DJ, Price EV, Gill S, Javaid S, Coletti ME, Jones VL, Bodycombe NE *et al*: **Correlating chemical sensitivity and basal gene expression reveals mechanism of action**. *Nat Chem Biol* 2016, **12**(2):109-116.
